# 2D Hetero-Nanoconstructs of Black Phosphorus for Breast Cancer Theragnosis: Technological Advancements

**DOI:** 10.3390/bios12111009

**Published:** 2022-11-11

**Authors:** Soji Soman, Sanjay Kulkarni, Abhijeet Pandey, Namdev Dhas, Suresh Subramanian, Archana Mukherjee, Srinivas Mutalik

**Affiliations:** 1 Department of Pharmaceutics, Manipal College of Pharmaceutical Sciences, Manipal Academy of Higher Education, Manipal 576104, Karnataka, India; 2 Radiopharmaceuticals Division, Bhabha Atomic Research Centre, Mumbai 400085, Maharashtra, India

**Keywords:** black phosphorous nanomaterials, nanotheranostics, breast cancer, phototherapy, targeting

## Abstract

As per global cancer statistics of 2020, female breast cancer is the most commonly diagnosed cancer and also the foremost cause of cancer death in women. Traditional treatments include a number of negative effects, making it necessary to investigate novel smart drug delivery methods and identify new therapeutic approaches. Efforts for developing novel strategies for breast cancer therapy are being devised worldwide by various research groups. Currently, two-dimensional black phosphorus nanosheets (BPNSs) have attracted considerable attention and are best suited for theranostic nanomedicine. Particularly, their characteristics, including drug loading efficacy, biocompatibility, optical, thermal, electrical, and phototherapeutic characteristics, support their growing demand as a potential substitute for graphene-based nanomaterials in biomedical applications. In this review, we have explained different platforms of BP nanomaterials for breast cancer management, their structures, functionalization approaches, and general methods of synthesis. Various characteristics of BP nanomaterials that make them suitable for cancer therapy and diagnosis, such as large surface area, nontoxicity, solubility, biodegradability, and excellent near-infrared (NIR) absorption capability, are discussed in the later sections. Next, we summarize targeting approaches using various strategies for effective therapy with BP nanoplatforms. Then, we describe applications of BP nanomaterials for breast cancer treatment, which include drug delivery, codelivery of drugs, photodynamic therapy, photothermal therapy, combined therapy, gene therapy, immunotherapy, and multidrug resistance reversal strategy. Finally, the present challenges and future aspects of BP nanomaterials are discussed.

## 1. Introduction

Cancer, which is caused by the irregular and uncontrolled growth of cells due to hereditary or induced genetic impairment, is the most important root cause of death worldwide after heart diseases [1,2,3]. Among the clinically important human cancers, breast cancer is the most frequent cancer in women globally [4,5,6]. For the past three decades, the rate of incidence of breast cancer has increased by 3.1% worldwide. It is the major type of cancer causing deaths among women in developed and developing countries [7,8]. Globally in 2018, from 18.08 million new incidences of cancer, 2.08 million cases were breast cancer (incidence rate of 11.6%), and in 9.55 million deaths related to cancer, 626,679 deaths (about 6.6%) were implicated by breast cancer [9]. Two major targets in breast cancer genesis are estrogen receptor α (ERα) and epidermal growth factor 2 (HER2/ERBB2). The expression of ERα can be observed in almost 70% of breast cancers. When ERα is activated with estrogen, the oncogenic growth pathway in breast cancer cells becomes activated. Hormones can be used to downregulate the ER signaling. ERBB2 is overexpressed in 20% of total breast cancers. It is a transmembrane receptor from the family of epidermal growth factor receptors. Triple-negative breast cancer is another type of breast cancer characterized by the lack of expression of targets, such as progesterone receptor (PR), ER, or ERBB2; It makes up approximately 15% of all breast cancers [10,11]. Chemotherapeutic agents used for breast and other cancers are mostly hydrophobic and, thus, have poor aqueous solubility and bioavailability [12,13,14]. The major limitations of conventional therapies include collateral damage from nonspecific targeting by chemotherapeutic agents and rapid clearance from the circulation due to macrophage action. This minimizes the interaction between the drug and the cancerous cells, rendering them therapeutically ineffective. Multidrug resistance is another drawback of the conventional strategies [15,16].

Nanotheranostics is a more recent and promising approach in personalized medicine. It is developed by incorporating therapeutic and diagnostic moieties in a single nanoplatform. The usage of nanotheranostics permits the detection of the targets, noninvasive tracking of the drug distribution, assessment of therapeutic responses, and optimization of individualized treatment, thus enhancing treatment efficacy and the safety of patients [17,18]. It can potentially overcome most drawbacks of conventional therapy for breast cancer. Two-dimensional (2D) materials have recently gained wide interest owing to their distinct electronic and optical characteristics. Two-dimensional materials prepared using graphene, molybdenum, and tungsten have been explored extensively in the biomedical field for multiple therapeutic and imaging applications. The inherent optical characteristic of these materials makes them a potential platform for the theranostic application. However, even the above-referred categories of 2D nanomaterials may show poor uptake and high tissue toxicity, or lack sufficient surface area to anchor higher amounts of imaging agents or antitumor agents [19].

These limitations can be overcome by using 2D materials constructed from black phosphorus (BP). Black phosphorus nanosheets (BPNSs) are an advanced type of 2D materials that show outstanding optoelectronic properties and have extensive applications in the biomedical field. Since BP is a remarkable option with great optical characteristics and drug loading capability, it has been used for the management of a variety of cancer types, which include: osteosarcoma [20,21], liver [22], lung [23], melanoma [24], breast [25], and cervical [26] tumors. This review focuses on the use of BP nanomaterials for the imaging and therapy of breast cancer, as the literature update on BP’s application in breast cancer management is rare. There is a need for an up-to-date and thorough critical review on of BP’s wide spectrum theragnostic applications in breast cancer in the light of a growing demand for BP as a substitute for graphene-based materials. Furthermore, various physiochemical features, synthesis strategies, and surface modifications of BP will be discussed in order to provide a thorough overview of this unique material. A special focus has been placed on the therapeutic, imaging, and diagnostic platforms of BP for breast cancer. A comprehensive section is dedicated to the various therapeutic strategies for the management of breast cancer, which includes drug delivery, codelivery of drugs, gene therapy, sonodynamic therapy, photothermal therapy (PTT), photodynamic therapy (PDT), and immunotherapy. The toxicity, biodegradation, and future perspectives of black phosphorous nanostructures are also briefed.

## 2. Black Phosphorus Platforms in Breast Cancer

The nanoplatforms of BP can broadly be classified as 0-dimensional (quantum dots; BPQDs), 1-dimensional (nano-/microbelts, nanoribbons) [27], 2-dimensional (nanosheets), and 3-dimensional nanoparticles (NPs) (sponges) [28], as well as other formulations, such as hydrogels and nanocomposites [29]. Due to their confinement in different dimensionalities, nanocrystals have different bandgap energies, and this bandgap is a crucial element for quantitative alteration via regulating the composition, size, and dimensionality. Despite having the same inner bonding structure, the optoelectronic properties of the nanostructures change with size because of the changes in the density of energy levels [30]. This enables researchers to employ BP as different nanostructures based on the desired application. Among the different types of BP nanomaterials, nanosheets are the most widely explored due to their efficient drug loading capacity, which is mainly ascribed to the large surface area. Similar to other BP nanomaterials, nanosheets also have good biocompatibility, biodegradability, and photostability. They also have demonstrated an excellent ability to produce singlet oxygen photosensitizers and are, therefore, widely used in phototherapies. Furthermore, several studies have been reported on the development of surface-modified BPNS platforms for biomedical applications, including breast cancer [31,32]. After nanosheets, BP quantum dots (QDs) are the next most widely studied nanoplatform for the therapy of breast cancer. They exhibit good tissue penetration and biological imaging functionality, along with the features of phototherapy. BPQDs having a particle size in the range of 10 to 20 nm are more likely to be distributed in the tumor tissues because of their ability to elude macrophages, and thus have a longer circulation half-life. Further, due to the enhanced permeation and retention (EPR) activity, they show preferential accumulation of antitumor drugs at the tumor site. Additionally, BP degradation is amplified under low pH conditions, improving the release of antitumor drugs in an acidic tumor microenvironment [33]. As a result, BPQDs are nearly nontoxic and have beneficial characteristics, including enhanced biocompatibility, desired degradability, and efficient therapeutic potential. Other nanostructures of BP, such as nanoribbons, have found applications in biomolecular sensing and/or DNA nucleobase identification [34,35] and are reported to exhibit lesser degradation on exposure to water in comparison with nanosheets [36]. However, their application for breast cancer is not yet explored.

## 3. General Methods of Synthesis

The interlayer van der Waals force present in BP nanomaterials is weak in nature, which makes easier conversion of bulk BP to BPNSs [37,38,39]. Here, we review the current methods to prepare BP nanomaterials

### 3.1. Top–Down Methods

The method involves mechanical liquid-phase exfoliation, which affects interlayer noncovalent van der Waals forces. This method can effectively cleave bulk BP to monolayers. N-methyl-2-pyrrolidone (NMP) is an organic solvent that is used as an exfoliation agent with better efficacy than other organic solvents, such as ethanol and methanol. The sonication process accelerates the exfoliation efficiency of NMP [40,41,42]. Phytic acid has copious polar phosphorus as well as hydroxyl functional groups, which assist and accelerate exfoliation. The electrical, optical, and thermal characteristics of BPNSs depend on their number of layers and thickness [43]. In order to achieve high penetration or accumulation into tumor tissue, efficient drug delivery, and fast excretion, the particle size of BP nanomaterials should be small. To achieve that, ultrasmall BP QDs can be prepared by a top–down strategy. QDs were prepared with a two-step process, which includes ultrasound probe sonication, followed by ice-bath sonication with NMP [44,45].

**Figure 1:**
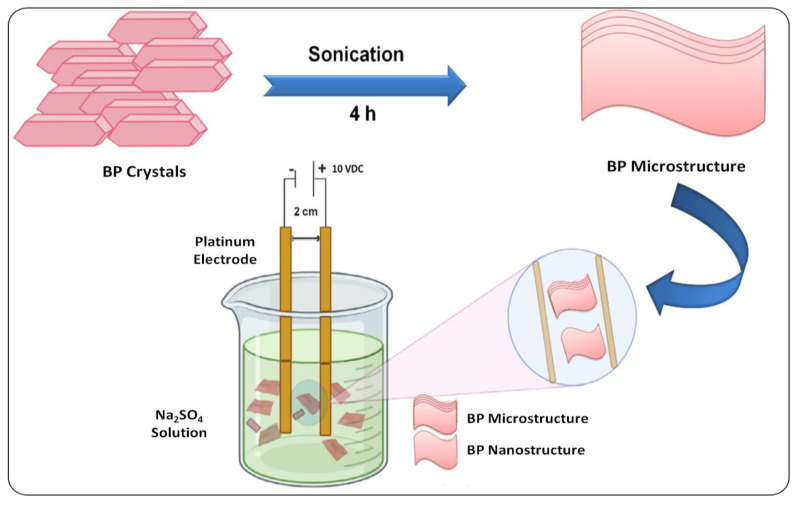
Synthesis of BP nanostructures via electrochemical exfoliation technique.

While using organic solvents for exfoliation, there is a chance of deposition of superfluous solvents on the surface of BP nanomaterials. This can be overcome by performing the exfoliation process in water. However, BP nanomaterials are less stable and highly active in aqueous solutions, which can lead to the degradation of these nanomaterials in water [46]. For this purpose, researchers have attempted to modify the surface in order to increase their stability, and have discovered that functionalization enhances their aqueous stability along with photothermal conversion efficiency [47,48]. A recent alternative approach used bipolar electrodes, where a potential difference was applied on opposite sides of bulk BP, resulting in its fractionation into BP nanostructures (Figure 1) [49]. In another study, the ionic liquid exfoliation technique was used for the synthesis of BPNSs. This involves grinding of bulk BP with ionic liquids, followed by ice-bath sonication [50]. Another promising approach is the plasma-assisted process, where monolayer BP was produced via mechanical cleavage continued by a successive Ar^+^ (Argon) plasma thinning procedure. The combination of the plasma-assisted process with exfoliation could control the thickness of BPNS [51].

### 3.2. Bottom–Up Methods

The chemical vapor deposition (CVD) method is broadly employed for the large-scale synthesis of many 2D nanomaterials, such as transition metal dichalcogenides (TMDCs) and graphene. However, the fragile nature of BP on exposure to air and lack of any appropriate substrate suitable for CVD growth make these materials inappropriate for the CVD technique [52,53]. The preparation of BP from red phosphorus (RP) directly on a silicon substrate was reported by Joshua et al. Raman spectroscopy and transmission electron microscopy (TEM) confirmed the successful conversion of RP to BP [54]. There are reports on the BP synthesis from RP using Cu as a catalyst, along with SnI_4_ and Sn. A mixture of copper, tin, RP, and SnI_4_ was taken in a silica glass ampoule. After that, the ampoule was vacuum-sealed and maintained horizontally on the hot end of the oven for 4 h at 923 K. The reaction mixture was then cooled back to room temperature within three days at a cooling rate of 0.2 °C/min. BP started to crystallize, and excess SnI_4_ was removed with sonication in an ultrasonic bath for 15 min, followed by refluxing of the mixture in toluene. To confirm the purity of the prepared BP, X-ray diffraction (XRD) and energy dispersive X-ray (EDX) analysis were performed [55].

**Figure 2:**
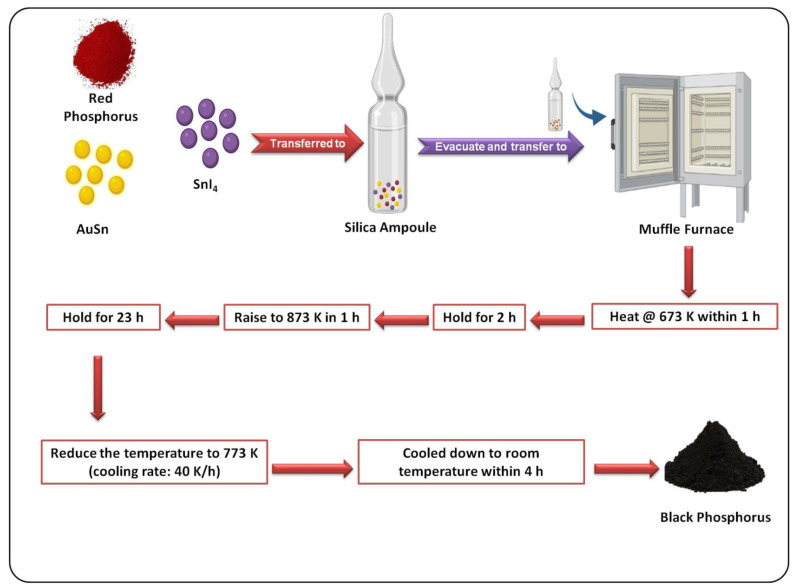
Facile synthesis method of black phosphorus.

A facile method to prepare BP from RP and gold--lambda~2~-stannane (AuSn) was fabricated by Nilges et al., as shown in Figure 2. It was found to be a more efficient method to prepare BP than conventional bismuth flux and high-pressure techniques [56]. In a recent work, a high-energy mechanical milling method using a ball mill for preparing BP from RP was introduced. XRD along with high-resolution transmission electron microscopy (HRTEM) was performed to confirm the formation of BP. In the HRTEM image, there were several edge dislocations, revealing that mechanical milling transformed the amorphous RP into BP nanocrystals. Under the influence of a huge number of edge dislocations in the crystals, the grains were refined and turned into small particles. The ball milling procedure is nontoxic, easy, and effective when compared with other methodologies [57].

BP preparation by rapid one-step microwave-assisted liquid-phase synthesis (MLS) was reported for the first time by He et al. BP is produced in situ on RP to produce a BP–RP heterostructure, which acts as a metal-free photocatalyst that may be employed directly. The structural conversion from a spherical to a layered form of BP was confirmed with field-emission scanning electron microscopy. With BP functioning as a cocatalyst, the coupled BP–RP heterostructure showed higher photocatalytic degradation of methylene blue by visible light. The developed MLS approach has a lot of potential for creating a quick and effective way to prepare BP for photothermal applications [58].

## 4. Characteristics of BP Nanomaterials for Breast Cancer Theranostics

**Figure 3:**
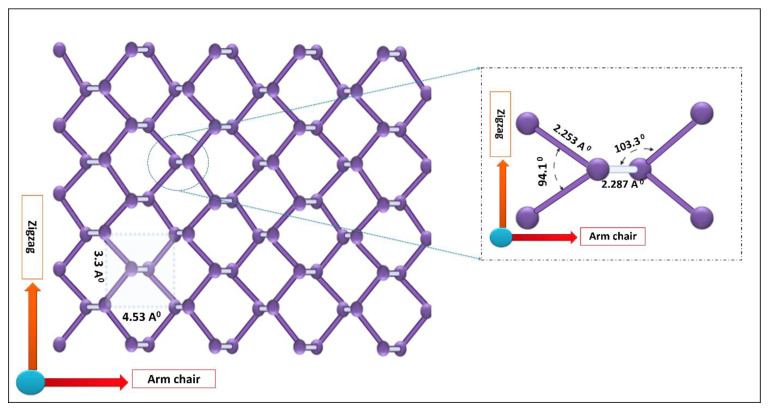
Schematic illustration of the structure of black phosphorus.

BP nanomaterials exhibit a puckered honeycomb structure, wherein each phosphorus atom is sp^3^ hybridized with a tetrahedral arrangement, as shown in Figure 3. Due to this arrangement, BP nanomaterials show outstanding optoelectronic, thermal, and mechanical properties [59], which result in its extensive application in the biomedical field, as summarized in Table 1. In this section, we have described how these individual properties of BP have a role in cancer treatment. BP nanomaterials have strong optical absorption, a very good NIR extinction coefficient, and an adjustable bandgap. This enables them to act as photosensitizers or photothermal agents, wherein they convert the energy of incident light (NIR irradiation) to heat, resulting in photothermal ablation of tumors [19]. Moreover, this energy obtained after excitation of BP nanomaterials can also get transferred to the surrounding oxygen and lead to the evolution of singlet oxygen or reactive oxygen species (ROS), which play a vital part in the photodynamic therapy of the tumor [60]. Due to their honeycomb structure, BP nanoplatforms have corrugated textural and crystalline properties, which provide them with a great “surface-area-to-volume ratio” for loading of active therapeutics with high efficiency [61]. For instance, when doxorubicin (DOX) was loaded onto BPNS through electrostatic interaction, the loading efficiency was 950% in weight when the concentration of DOX was 1 mg·mL^−1^, remarkably higher than with previously reported methods [62]. The biocompatibility and biodegradability of BP can be ascribed to the fact that phosphorus accounts for almost 1% of the human body weight and is a fundamental element connected with the formation of deoxyribonucleic acid (DNA), mineral components of the hard tissue, and the cell membranes [63]. As described in the earlier sections, BP tends to degrade into P_x_O_y_ species after contact with oxygen and water. The degradation products of BP are nontoxic phosphates, which trap the calcium ions, leading to the production of calcium phosphate, a component of bone regeneration. In addition, when the cytosolic levels of these phosphates are elevated, it causes oxidative stress, retarding the proliferation of tumor cells and result in the induction of apoptosis [64].

## 5. Surface Modification of BPNSs

In the earlier section, we discussed the various possibilities of synthesizing BPNSs. However, the bare nanoplatforms of BP have stability issues when exposed to the external environment. They get easily oxidized in the presence of water and oxygen to form P_x_O_y_ products of phosphorus. When BP comes in contact with oxygen, either it gets oxidized into chemisorbed phosphorene, or there is a possibility for the introduction of oxygen interstitially, hampering the purity and anisotropic properties. Furthermore, due to the affinity of BP to water, it is easily degraded [33]. Surface modification is reported to be effective in overcoming these issues. It not only helps in enhancing the stability of the BPNS, but also introduces other features, such as targeting ability, functionalization, and improvement of drug loading efficiency. Modification can be enabled by either electrostatic interaction or chemical conjugation.

The most commonly used and reported groups for this purpose are polyethylene glycol (PEG), poly(2-ethyl-2-oxazoline) (PEOz), and amino compounds, such as polyethylenimine (PEI), human serum albumin (HSA), and polydopamine (PDA) [72]. For instance, the modification of BP with PEG-NH_2_ was performed in one of the approaches using the principle of electrostatic adsorption. The authors reported that the stability of BP was enhanced to a great extent, with almost negligible degradation after modification, as observed through the Tyndall effect and UV–VIS absorption spectra. The abundant availability of NH_2_ groups after modification enhanced the drug delivery ability [26,73,74]. Similar to PEG-NH_2_, PEI has abundant -NH_2_ groups, which help to neutralize the negative charge. Many researchers have reported the use of PEI for surface modification of BP to boost stability. PEI could also protect siRNA upon adsorption and prevent its enzymatic degradation. It has also been used for the functionalization of negatively charged gold NPs. Another widely used molecule for the surface modification is PDA, a biomimetic polymer with excellent adhesive capacity. The amino groups present on the PDA enable covalent linkage of the functional moieties via chemical conjugation reactions, such as carbodiimide chemistry with the carboxyl groups from triphenyl phosphonium (TPP) and chlorin e6 (Ce6), and reactions with BTZ [19,75]. PDA also has good photothermal effects and can enhance the stability of BPNS. Several other approaches are available for surface modification of BPNS, as described in detail elsewhere [33,72,76]. Here in this article, we have categorized the possibilities of surface modification into stimuli-responsive modifications and cell-organelle-targeted modifications.

### 5.1. Stimuli-Responsive Modifications

A substantial amount of research has gone into developing stimuli-responsive drug release. The design of an efficient stimuli-responsive delivery system for regulated drug release is driven by the lesions and differing cellular environments among cancer and normal cells, such as blood vessels, pH, and ROS. These new systems can be programmed to react to pH, enzyme activity, light, ultrasound, and heat. Some systems are even sensitive to a combination of two or more stimuli. To date, many functionalization approaches have been implemented for the development of a BP-based stimuli-responsive delivery system [77].

#### 5.1.1. Functionalization for pH-Responsive Drug Release

pH-responsive delivery systems have attracted the most attention among the numerous BP-based stimuli-responsive systems. Substantial pH fluctuations in several parts of the human body benefit the usage of stimuli-responsive systems [78]. Some anticancer agents, such as DOX, are insoluble; however, pH-responsive delivery systems can bypass this limitation by protonating the drug via NH_2_. Luo et al. constructed PEGylated BP for the delivery of DOX. Drug release studies have suggested that at a pH of 7.4, about 16.4% of drug was released, while at pH 5.0, it was 37.8%. This difference in drug release could be attributed to the enhanced protonation of the amino group present in DOX [79]. The pH-stimulative release of the anticancer drug mitoxantrone (MTX) has also been investigated; pH 5.0 showed a higher release of about 1.25-fold than that of 7.4 pH, owing to the amino group protonation of MTX molecules in the acidic condition [80]. Gao et al. fabricated a BP-based nanocarrier loaded with DOX and functionalized with PEOz. It has a pKa that is comparable to physiological pH and may be changed by altering the molecular weight. PEOz charge is changed from negative to positive at a pH lower than its pKa by tertiary amide group ionization on the PEOz chain. A PEOz-functionalized drug nanodelivery system may be enriched and charge-reversed in the weakly acidic environment of tumor tissue at pH of 6.8, permitting endocytosis and pH-responsive drug release after being stimulated by endosomes and lysosomes’ low pH of 5.0 [19]. These pH-activated delivery systems exhibit excellent drug solubility, higher cellular uptake, and improved effective drug delivery.

Polydopamine (PDA) has been extensively researched as an important coating layer. A PDA “capsule” coated on drug-loaded BP NSs has been found to successfully control sudden drug release bursts. Controlled release of anticancer drugs is preferred for efficient cancer treatment. More significantly, the pH sensitivity of the PDA coating causes its dissociation from the surface in acidic environments [81]. Wu et al. evaluated the DOX release characteristics in different pH environments from PDA-coated BP nanoformulation. The cumulative drug release from the BP nanosystem at different pH’s of 7.4, 6.8, and 5.0 was 11.2%, 17.7%, and 31.8%, respectively [82]. Gao et al. examined the drug release of BP nanoformulation and observed 11.3% of drug release at 7.4 pH and 29.3% at a pH of 5.0 over 36 h [19]. As a result, the surface coating with PDA on BP worked as a protective capsule, regulating DOX release at the tumor region.

#### 5.1.2. Functionalization for Light-Responsive Drug Delivery

Light irradiation has received substantial interest as a noninvasive technique for remote spatiotemporal control of drug release at the required place and time, among the other stimuli employed in smart delivery systems [83]. Shin et al. developed a multifunctional poly (N-isopropylacrylamide) (PNIPAM) nanocomposite for synergistic chemo-photothermal treatment against breast cancer cells [84]. Acrylic acid (AA) was added to the PNIPAM to enhance the transition temperature, resulting in a shift in the intrinsic lower critical solution temperature to 42 °C. Polypyrrole NPs were evenly coated with the PNIPAM-AA complex to produce the photothermal effect under an NIR light irradiation of 808 nm. FA was effectively conjugated on the excess carboxyl groups in the PNIPAM network as a cancer-targeting ligand. The DOX release of PNIPAM-AA-FA nanoconjugates was effectively activated by NIR laser irradiation in response to temperature variation. They also discovered that PNIPAM-AA-FA was internalized by folate-receptor-mediated endocytosis in MDA-MB-231 breast cancer cells, significantly improving cancer therapy efficacy with chemo-photothermal effects.

### 5.2. Cell-Organelle-Targeted Modifications

Targeting cell organelles renders several benefits in the treatment and imaging of cancer cells. Due to the acidic pH in cancer cells, a considerable pH gradient within the different organelles (cytosol (7.4), Golgi body (6.4), endosomes (5.5–6), and lysosomes (4.5–5)) [72] encourages researchers in developing pH-responsive nanoplatforms, which can deliver active moieties to specific organelles. When it comes to the targeting of cellular organelles, the most explored organelle is mitochondria, which play a prominent role in the life and death of cells. In addition to mediating apoptosis and being referred to as the “powerhouse of the cell”, mitochondria are also in charge of cellular respiration and have been found to be a valuable target for the effective destruction of cancer cells [85]. When compared with other organelles, mitochondria are found to be more sensitive to heat and exhibit an increased transmembrane potential in cancer cells, forming an effective target for photothermal therapy. Several molecules, such as triphenylphosphine (TPP), triethylammonium pyridinium, and guanidinium, are widely explored for the targeting of mitochondria [86]. Utilizing this targeting property of lipophilic cations, a research team [87] developed a heterobifunctional molecule (PEG and TPP) and grafted it on BPQDs via a covalent bond. The polymer grafting enhanced the dispersibility and stability, and imparted mitochondria-targeting ability to the BPQDs under physiological conditions. The results of in vitro studies performed on a breast cancer cell line (MCF-7) showed that the surface-modified NPs exhibited superior cytotoxicity to cancer cells. Meanwhile, antitumor efficacy studies performed on the tumor-bearing mice confirmed the efficient accumulation of NPs in tumors, leading to photothermal tumor ablation [87]. Similarly, in another study [88], a photoacoustic (PA) therapeutic platform of BPNSs was developed and modified with TPP and other immune adjuvants. Through the in vitro and in vivo studies performed on the breast cancer models, it was proved that the TPP enabled mitochondria targeting, the PA shockwaves generated after pulsed laser irradiation destroyed mitochondria, and the immune adjuvants promoted antitumor immune responses. Other organelles targeted BP-based nanoplatforms may also be developed and reported in near future, which would enhance the cancer targeting and therapy to a greater extent.

## 6. Modifications for Targeting

Specific targeting of tumor cells is envisaged by using nanoplatforms for anticancer drug delivery. Targeting helps to improve drug efficacy while reducing collateral damage to nontarget tissue. There are several reports on the construction of a BP-based targeted delivery of drugs. The targeting approaches can be classified into active and passive targeting, as detailed below [89,90].

### 6.1. Active Targeting

Active targeting is achieved by interaction of ligands with respective receptors expressed on tumor cells. Suitably chosen ligands on the surface of BP nanoformulations can target specific molecules overexpressed on the tumor cell surface. The receptor–ligand interaction instigates receptor-mediated endocytosis, which causes internalization of nanoformulation and release of the drug chemically bound to the BP nanosystem. In this manner, specific receptors on breast cancer cells can be induced to selectively take in therapeutic drugs for a curative effect [91].

#### 6.1.1. Folic Acid (FA)

Cost-effectiveness, excellent stability over different pH values and temperatures, precise targeting, and nontoxic characteristics make FA an ideal candidate for breast tumor targeting [92]. The attachment of FA to the folate receptors positioned inside the caveolae results in the internalization of a complex via the endocytotic pathway. The drug is released when the endosome pH reaches five by the dissociation of FA from its receptor [93]. The interaction between the folate receptor and FA can be utilized for breast cancer imaging by attaching imaging probes to FA or its analogues for targeting [94,95]. However, the folate receptor expression differed among breast tumor subtypes; triple negative/basal tumors had considerably higher folate receptor mRNA expression than ER+ and HER2+ cancers [96]. In a recent study by Deng and his team, they functionalized and formulated small BPNPs for the photothermal treatment and targeted imaging of cancer. They used FA and cyanine 7 for the functionalization of nanomaterials and employed PEI branching and dextran modification. After FA conjugation, a reduction in zeta potential was observed from +20.0 to +7.3 mV, where it was shifted to −24.5 mV on attachment with sulfo-cyanine7 NHS ester. FA-conjugated NPs showed 3.1-fold signal intensity and NPs devoid of FA showed 2.4-fold signal intensity compared with that of a precontrast image. The results indicated better targeting efficiency of an FA-conjugated BP nanosystem along with the EPR effect. The formulated BPNP system showed a nano-theranostic image-guided treatment (PTT) of breast cancer [97]. In another report, BPQDs were functionalized with FA and loaded with DOX for chemo-photothermal cancer treatment. The in vivo combined therapeutic efficiency study showed that FA-conjugated NPs exhibited better active targeting capability than NPs without FA. Photothermal evaluation studies demonstrated the ability of the system to be used as a PTT agent and exhibited good targeting efficacy for tumor killing [79]. Researchers are widely using FA as a targeting ligand to conjugate with BP nanomaterials to improve the recognition capability, targeted drug delivery, and efficient uptake of BP nanomaterials by breast cancer cells.

#### 6.1.2. Hyaluronic Acid (HA)

HA is an essential constituent of the extracellular matrix (ECM), derived from natural mucopolysaccharide made up of N-acetyl-D-glucosamine and D-glucuronic acid as repeating units [15,98]. HA receptors are overexpressed in tumor cells, which include a group of cluster of differentiation 44 (CD44) receptors and the receptor for hyaluronic-acid-mediated motility (RHAMM) [99]. Selective tumor targeting is possible via HA conjugation, and it will also enhance the stability of the anticancer drug in the formulation. In a recent study, Zhang and team formulated an NIR- and pH-responsive drug delivery system for combinational chemo-/photothermal therapy of cancer with BPNSs. The conjugation of HA resulted in a significant difference in zeta potential from −32 to −18 mV. Charge reversal, red shift, and notable change in particle size confirmed MTX loading and HA surface conjugation to the BP nanomaterial. It was observed that modification with HA caused an increase in tumor microenvironment temperature compared with bare BP. The formulation showed good pH/NIR laser (808 nm)-induced drug release with a temperature rise to 45 °C in 3 min. It precisely targeted tumors with the HA moiety in addition to the EPR effect [80].

There are reports on HA-conjugated BPNSs loaded in a poly (amidoamine) (PAMAM) dendrimer for chemo-/photothermal breast cancer therapy. Because of nonspecific interactions with both normal and malignant cells, positively charged NH_2_ groups in the periphery of the PAMAM dendrimer can lead to significant cytotoxicity and hemolytic toxicity. The addition of HA to the nanosystem caused an effective reduction in toxicity by masking positive surface potentials. The presence of PAMAM and the HA layer improved the stability of BPNSs. The prepared nanosystem showed excellent NIR and pH simulative drug-releasing characteristics. In vivo and in vitro studies proved that the formulation exhibited greater targeting to the tumor cells [100]. Poudel and coworkers synthesized BPNSs from RP by the batch-by-batch route technique and utilized them as a nanoplatform for the delivery of DOX using HA as the targeting ligand molecule. Targeted delivery of the system to the CD44-overexpressing tumor cells was confirmed by its NIR-induced photothermal property [101].

#### 6.1.3. Targeting with Cell Membrane Coating

Natural cell membranes are the most biocompatible vectors for surface functionalization of NPs. The coated NPs will acquire the inherent physicochemical properties of cell membranes [102,103].

##### Erythrocyte Membrane

**Figure 4:**
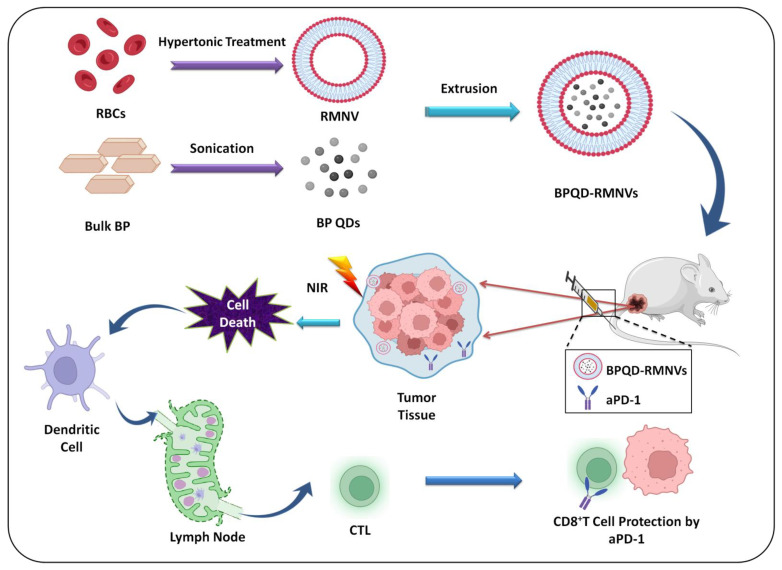
Diagrammatic representation of synthesis and photothermal tumor immunotherapy shown by BPQD-RMNVs and aPD-1.

The erythrocyte membrane can act as an efficient carrier of different drugs, drug delivery platforms, enzymes, proteins, and other macromolecules [104]. Erythrocyte-based drug delivery systems show excellent protection from a variety of endogenous factors, controlled release, and targeted delivery of chemotherapeutics; they have also wide applications in diagnostic and biomedical sciences [105]. Recently, Liang and team formulated a biomimetic BPQD system, which is surface-coated with an erythrocyte membrane (RM) for NIR-irradiation-induced triple-negative breast cancer cell apoptosis and attached with a PD-1 antibody to eliminate metastatic and residual tumor cells (Figure 4). The in vivo potential of this BPQD formulation was evaluated in 4T1-tumor-bearing mice. Comparison of tumor in situ temperatures after NIR irradiation demonstrated that the PTT efficiency of membrane-coated BPQDs was more than that of bare BPQDs. The administration of erythrocyte-membrane-coated BPQD-mediated PTT successfully inhibited the primary and secondary cancer cell growth, indicating excellent potential for clinical therapy of breast cancer [83].

##### Platelet Membrane

Platelets are blood cells that assist in clotting. Coating of the platelet membrane (PLTM) to drug delivery systems can help the formulation to escape macrophage uptake and significantly enhance circulation half-life [106]. As the PLTM expresses certain surface receptors, such as P-selectin and glycoprotein Ib (GPIb), it can also actively adhere to the tumor cells and damaged vasculature [107]. PLTM-coated BPQDs loaded with hederagenin (HED) and labeled with Cy5 were prepared by Shang and his coworkers in 2019, where they could efficiently target the tumor cells and potentiate the antitumor activity. Intravenously injected PLTM@BPQD-Cy5 showed higher fluorescence intensity than BPQD-Cy5, which confirmed greater accumulation with an improved selectivity of PLTM@BPQDs-Cy5 in the tumor region as compared with uncoated BPQDs-Cy5. After 48 h of injection, retention of PLTM@BPQD-Cy5 was more than that of BPQD-Cy5, while kidney and liver accumulation of PLTM@BPQD-Cy5 was less than BPQD-Cy5. These results suggest that PLTM@BPQDs are more efficient in the precise delivery of a chemotherapeutic agent with excellent targeting capabilities [108].

##### Neutrophil Membrane

Neutrophil membrane (NE) camouflaging NPs offer a novel strategy for attaining targeted therapeutic administration in the cancer and inflammation therapy. The NE was isolated and purified to coat the surface of NPs in the cell membrane biomimetic nanosystem. The generated biomimetic NPs can inherit the biological activity of the source cells and perform the role of intercellular communication [109]. The neutrophil (NE) membrane was coated with BP nanoflakes combined with the transforming growth factor-β (TGF-β) inhibitor. BP’s PDT and PTT caused acute local inflammation in the tumor and enhanced nanoparticle accumulation via NE-membrane-mediated affinity. PDT and PTT in combination with a TGF-β inhibitor caused strong immune activation in the tumor and significantly reduced cancer metastasis. The results showed that the positive feedback mechanism aided combination treatment by providing greater anticancer efficacy than nonpositive feedback nanoformulations [110]. The biomimetic drug delivery system possesses an excellent stability profile, nonimmunogenicity, biodegradability, and flexibility, making it an efficient candidate for cancer therapy, gene therapy, and other medical applications.

#### 6.1.4. Antibodies

PD-1 antibody (aPD-1) treatment elicits a substantial response against basal-like breast cancer cell because of its ability for the reactivation of tumor-infiltrating CD8+ T cells,. In a study by Ling et al., they developed a biomimetic nanoformulation of erythrocyte-membrane-coated BPQD nanovesicles (BPQD-RMNV) to actuate triple-negative breast cancer apoptosis in situ on irradiation with NIR light. They combined this strategy with the attachment of aPD-1 to activate the immune system and get rid of cancer cells that had spread to other parts of the body. Dendritic cells (DCs) were recruited, and they captured the released neoantigens as a result of NIR-light-induced tumor cell apoptosis. Consequently, the primary and secondary tumors were significantly stimulated by tumor-specific CD8^+^ T lymphocytes. In vivo studies on 4T1-cell injected BALB/c mice demonstrated that the inclusion of aPD-1 revived the CD8^+^ T cells from collapse to find and destroy the metastatic and remaining tumor cells [83]. Zhao et al. developed a nanoformulation of BPQDs attached with an anti-PD-L1 antibody. In this study, biomimetic BPQDs with cancer cell membrane coatings were created for tumor-targeted PTT. They discovered that biomimetic BPQDs had a high photothermal conversion efficiency, which has the ability to not only directly destroy tumors via photothermal effects, but also to trigger dendritic cell maturation. Additionally, PD-1/PD-L1 pathways are blocked by anti-PD-L1 therapy, which also improves the T-cell immune response. This makes it possible for T cells to identify and eliminate tumor cells. Combining biomimetic BPQD-mediated PTT with anti-PD-L1 immunotherapy prevents tumor recurrence and metastasis by converting the tumor microenvironment into an immune-active microenvironment, which strengthens both the local and systemic anticancer immune response. In vivo investigations in BALB/c mice bearing 4T1 tumor have shown that the combination photothermal immunotherapy method is more effective against tumors than anti-PD-L1 monotherapy. They also demonstrated that this combined therapy technique has an immunological memory effect that protects against tumor recurrence. This study indicates that the combination a photothermal immunotherapy technique increases TNBC effectiveness, efficiently suppresses recurrence and metastasis, and offers a wide variety of cancer therapeutic applications [111].

#### 6.1.5. Peptides

Peptides have gained popularity as tumor-targeting ligands as a substitute for antibodies and affibodies. Peptide-based therapies have several benefits. Their small size permits for simple penetration to deeper tissues, they may be less immunogenic, and they may be easily synthesized utilizing automated solid phase techniques, allowing for quick synthesis with high yields and at a cheap cost [112]. RGD is a type of short peptide that includes arginine–glycine–aspartate (Arg–Gly–Asp) that may particularly target integrin receptor-like ανβ3, which is largely expressed on the cancer cell surface and neovascular endothelial cells. Zhong et al. formulated an RGD-peptide-conjugated RBC membrane by the lipid insertion approach for an improved photothermal effect. In vitro targeting capacity studies showed an increase in the fluorescence intensity in RGD-conjugated formulation compared with RBC-BP and PEGylated BP, which confirmed significant targeting by the RGD peptide to the cancer cells. In vivo investigations revealed that RGD-RBC-BP nanoformulation extended circulation duration, more tumor accumulation and less uptake in the liver, and displayed significantly superior photothermal effectiveness in mice when compared with PEGylated BP [113]. Hu et al. proposed a strategy to enhance the photothermal stability of BP nanomaterials by conjugating them with Cu^2+^ because Cu ions can act as BP nanocaptors, Cu-based materials have efficient photothermal activity (PTA), and they have a synergistic effect on BP degradation and photothermal activity. An RGD-PEG composite was conjugated to BP@^64^Cu for enhanced tissue-specific localization and internalization. An in vivo tumor-targeting ability study of BP@Cu@PEG-RGD in an MDA-MB-231 tumor model showed the specific uptake by the tumors, despite the size of tumor. Furthermore, the therapeutic effectiveness of BP@Cu@PEG-RGD was validated by PET imaging, which revealed that animals treated with the formulation had smaller tumors than mice treated with saline [114].

The term “tumor homing peptides” (THPs) refers to linear/cyclic peptides of a few amino acids that naturally detect tumor cells and particularly attach to receptors on the tumor cells, blood arteries, or lymphatic vessels. These are crucial for effective cancer treatment because they may be used as a key tool for targeted medication delivery, particularly to malignant cells [115]. Beidulska et al. introduced a novel strategy for the functionalization and stabilization of few layer-BP (FL-BP). They used nonionic PEG and polylysine (PLL) polymer for the efficient encapsulation of FL-BP and to avoid environmental degradation. THPs were produced by 9-fuorenylmethoxycarbonyl chemistry. In the first step of conjugation, THPs were bioconjugated with PLL or a PEG linker, and in the next step, the conjugated peptides were noncovalently immobilized to the FL-BP surface to form FLBP-PEG/PLL-THP. In order to evaluate the cytotoxic effects of FLBP-PEG/PLL-THP, MDA-MB-231, MCF-7, and HB2 cell lines were administered with different concentrations of nanoformulation. The cytotoxicity of FLBP and its modifications evaluated with MTT assay indicated the cell and dose-dependent nature of nanoformulation. HB2 (normal mammary cells) showed toxicity only at a higher concentration of 20 µg/mL, whereas MCF-7 (69% viability) and MDA-MB-231 (38% viability) breast cancer cells started to lose viability at 4 µg/mL. These findings suggest that the functionalization of FLBP with PLL decreased its cytotoxicity in healthy mammary cells; it was toxic to malignant cells, particularly the triple-negative MDA-MB-231 breast cancer cells [116].

#### 6.1.6. Proteins

There are many studies going on in the area of protein-conjugated inorganic nanocarriers to fabricate multifunctional theranostic platforms. Yue and colleagues designed a novel platform of BP functionalized with PLL (PBP) for Cas13a/crRNA delivery for the therapy of breast cancer. An antiapoptotic protein called Mcl-1 has been shown to be a significant predictor of breast cancer, and thus, Mcl-1 mRNA is a suitable target for breast cancer treatment. They found that the constructed PBP@Cas13a/crMcl-1 nanosystem may suppress Mcl-1 at the transcriptional level, lowering Mcl-1 protein expression and increasing apoptosis in breast cancer cells through in vitro and in vivo tests [117]. In another study, Zhou and coworkers successfully loaded Cas9 ribonucleoprotein modified with three nuclear localization signals (NLSs) at the C-terminus (Cas9N3) into the BP nanosheet through electrostatic interaction for enhanced nuclear targeting and improved electrostatic interaction. The developed formulation could deliver BP-Cas9N3 to the cytoplasm by endocytosis and direct membrane penetration. The escape of the formulation from the endosome was facilitated via the biodegradation of the BP nanomaterial. Successful in vivo and in vitro gene silencing and genome editing were performed as NLSs directed Cas9N3 transportation to the nuclei. It exhibited better biodegradability and biocompatibility and can be considered a novel approach for personalized medicine and gene therapy [118].

### 6.2. Passive Targeting

Compared with normal tissue, cancer tissue has higher vascular wall gaps, has a larger number of blood vessels, is devoid of lymphatic circumfluence, and has imperfect structural integrity. These characteristics lead to selective increased uptake of drugs in tumor blood vessels, which is known as EPR effect. Entry of BP nanomaterials into the tumor microenvironment is by EPR-dependent passive targeting, which makes use of diffusion mechanisms and endocytosis to pass the cell membrane [119,120]. The unique features of tumor blood vessels and the size of BP nanomaterials are the two important parameters that influence circulation time. BP nanomaterials of particle size in the range of 20 to 200 nm bypass renal filtration, which causes enhanced passive accumulation of materials in tumor cells via the EPR effect. Shao and coworkers developed a biodegradable BP-based nanosphere for in vivo PTT. They loaded poly(lactic-co-glycolic acid) (PLGA) into BPQDs to formulate biodegradable BPQDs/PLGA nanosheets with an optimal size range of 127 ± 35.7 nm, which enabled improved accumulation and longer circulation time in tumor cells [63]. Wang and coworkers formulated a BP-based multimodal nanoagent for breast cancer by loading BPQDs with DOX and modifying with PLGA. In vitro and in vivo imaging studies indicated time-dependent localization of a BP-based nanoagent (~130 nm diameter) in tumor cells by the EPR effect [121]. In a recent study by Deng and his team, they functionalized BP NPs with FA and cyanine 7 for imaging and PTT of breast cancer. The formulation showed excellent potential for targeted imaging [97].

#### 6.2.1. pH-Sensitive Targeting

For cancer cells to become more invasive and to metastasize, they need refurbishing of the cell matrix and potentiation of enzyme activity, which demands an acidic environment. The pH in a normal tissue microenvironment is about 7.4, while in breast cancer tissues, it is reported to be between 5.4 and 7.1. This unique microenvironment with significant pH difference is harnessed by pH-sensitive targeting drug delivery systems [122]. Two important mechanisms were exploited for drug release from pH-sensitive drug delivery systems. One is by the protonation or deprotonation of carrier molecules by pH variation or hydrophobicity variation. The other is delivery of drug by pH-sensitive chemical bond cleavage. The pH-dependent drug release mechanism is safe and effective, as it is well reported to release the chemotherapeutic agent at the tumor site [123].

In a recent report, pH-responsive BP nanoformulation was formulated and modified with PEOz and PDA for chemo-/photothermal treatment of breast cancer. When PEOz comes in contact with an pH environment lower than its pKa, the negatively charged PEOz will become converted to a positively charged molecule via tertiary amide ionization on the PEOz chain. A lower pH of 5 at lysosomes and endosomes will then induce the drug release and endocytosis. Controlled drug release and improved tumor-targeting ability were observed by pH-sensitive targeting of drugs [19]. In another study, BPQDs were loaded with DOX and functionalized with FA-PEG-NH_2_. They performed a drug release study at different pH’s and NIR irradiations for 6 min several times. The developed formulation showed pH and photosensitive delivery characteristics, lowering potential harm to normal cells [79]. Zhang and team formulated NIR/pH dual-triggered BPNSs loaded with mitoxantrone hydrochloride (MTX) and coated with HA. The drug release experiment proved that MTX release was triggered both by NIR irradiation and pH simulation [80].

#### 6.2.2. Hypoxia-Mediated Targeting

Solid tumors possess regions with a reduced level of oxygen called tumor hypoxic regions. Tumor hypoxia is characterized by scarcity of oxygen and reduced nutrient supply to the internal regions of a tumor. The concentration of oxygen in this region ranges from 0.02%–2%, in comparison with 2%–9% in normal tissues. The enhanced anaerobic respiration, imbalanced angiogenesis, increased levels of ROS, and altered metabolism exhibited by the hypoxic tumor area make its environment acidic. This environment suppresses adaptive and innate immune response channels and results in the overexpression of different genes responsible for angiogenesis. As hypoxic tumor regions are known to show greater resistance to therapy and possibility of relapse, addressing hypoxia becomes necessary for effective tumor therapy [124,125]. The most common method for targeting the hypoxic microenvironment of tumors is by employing organic/inorganic materials having catalase-like activity, which converts H_2_O_2_ overproduced in breast tumor tissues into reactive oxygen species [126]. In a recent work, a bacterium/BPQD-conjugated system to increase the susceptibility of solid tumors to oxygen-dependent PDT was developed, which could target hypoxic cancer cells and achieved effective PDT. They attached catalase-expressing *Escherichia coli* bacteria with BPQDs via the electrostatic adsorption technique. The NIR laser irradiation of 660 nm destroyed the bacterial membrane and released catalase, which metabolized hydrogen peroxide to water and oxygen. The enhanced oxygen level attenuated intratumoral hypoxia, thereby enhancing the BPQD-mediated PDT [127].

In another study by Li and his colleagues, they developed PEG-functionalized BPQDs for combinational PDT-PTT. As the hypoxic tumor microenvironment causes reduced efficiency of PDT in breast cancer cells, they proposed an integration of PDT and PTT to overcome this limitation. The heat generated during PTT enhanced the blood flow, which resulted in improved oxygen supply and ^1^O_2_ production. PDT could also disturb the tumor microenvironmental conditions and potentiate the responsiveness of tumor cells to PTT [128]. A recent study proposed platinum nanoparticles (Pt NPs) as nanoenzymes for decomposing H_2_O_2_ as they exhibit good stability, desired capability for PDT, and facile functionalization. Pt NPs were attached to BPNSs through the reduction of chloroplatinic acid. By immunofluorescence staining of breast cancer tissues to assess the expression of hypoxia-inducible factor (HIF-1α), it was confirmed that Pt NPs could convert H_2_O_2_ to ROS for enhanced PDT. The combination of PDT and PTT resulted in enhanced therapeutic efficacy [129]. Drug resistance has been linked to hypoxia, and treating hypoxia in breast cancer patients is predicted to reverse drug resistance.

## 7. Imaging and Diagnosis

Noninvasive and rapid detection methods have been developed to identify cancers in the early stages. Different screening and imaging methods are available for early recognition of breast cancer. Screening methods, such as physical breast examination, mammography (film/digital), ultrasound scanning, and breast MRI, are employed as noninvasive detection methods [130,131]. Sentinel lymph node detection using radionuclides has an enormous potential in detecting the metastasis of cancer [132]. Further, sensitive and specific agents and imaging modalities are desired to overcome limitations of the existing methods. The four main techniques for imaging employing BP nanomaterials are described below.

### 7.1. Fluorescence Imaging

BP nanomaterials exhibit excellent optoelectronic property due to a layer-thickness-dependent bandgap. Interpretation of photoluminescence (PL) spectra reveals that thinner BP materials demonstrate a high intense PL peak [37]. In the case of layered BPNSs, bilayered nanosheets exhibit higher PL intensity than that of nanosheets with three to five layers [133]. Nile blue diazonium (NB-D) tetrafluoroborate salt dye was covalently conjugated into BPNSs to form NB/BPs, which can be detected with NIR fluorescence imaging with improved stability. The cell staining capability of the formulation was evaluated in breast cancer cells (MCF-7) and found to be suitable for fluorescent labeling of tumor cells. An in vivo fluorescence imaging study on MCF-7 breast tumor bearing BALB/c nude mice demonstrated a significant difference in fluorescence signals at different time intervals. Upon co-staining the formulation with Hoechst 33528 intense red fluorescent signals appeared in the cytoplasm of the cells, indicating its location of internalization. Bioimaging using the NIR fluorescence technique confirmed the efficient uptake of NB/BPs by the breast cancer cells due to EPR effects. The developed formulation can be explored as a potential platform for imaging of breast cancer [134].

BP nanomaterial coated with erythrocyte membrane (RM) for photothermal immunotherapy was formulated. The formulation was conjugated with Cy5.5 molecules to form Cy5.5/BPQD/RM. Fluorescence imaging was used to directly monitor the formulation’s in vivo tissue distribution in 4T1 tumor-bearing mice. Better accumulation of RM-loaded BPQDs was observed compared with bare BPQD. A fluorescence imaging study could confirm the elimination of formulation from the body in 24 h [83]. In another study, researchers explored NIR-II fluorescence of free-standing nanospheres of BP and their significance in NIR-II in vivo bioimaging. Imaging results revealed efficient spatial resolution, negligible autofluorescence, and better penetration compared with NIR fluorescence imaging [135]. BPNPs labeled with Cy5.5-NHS were formulated in another research to achieve photoacoustic-immune therapy. MCF-7 cells exhibited lower fluorescence intensity than that of 4T1 cells, which confirmed specific targeting of BP formulation to the 4T1 breast cancer cells [88].

PEGylated BPQDs were synthesized for bioimaging and photodynamic/photothermal synergistic breast cancer treatment. Confocal microscopic images gave intense fluorescent signals in 4T1 cells and HepG2 cells. High fluorescence intensity due to BP formulation was observed at the cytoplasmic region [128]. Similarly, carbon-dot-conjugated BPNSs were developed for the synergistic treatment of cancer. The in vivo fluorescence images were taken under 460 nm excitation, after intravenous injection of the formulations in animals bearing tumors. The observed fluorescent contrast confirmed the accumulation of nanosheets in the tumor region [136].

### 7.2. Thermal Imaging

As BP nanomaterials show outstanding photothermal conversion efficacy (PCE), the thermal image and temperature of the BP formulation under NIR irradiation can be assessed using thermal imaging devices in real time. BP nanomaterials are efficient thermal imaging agents since excellent in vivo thermal images have been obtained using the formulations [137]. Modification with polymers can further enhance the PCE of the nanosystem. A PDA modification approach was employed to enhance the stability and photothermal activity of the BP-based nanomaterial platform. Polymerization of dopamine produces PDA in a weakly alkaline environment; it is employed as an improved coating along with desired biodegradation and biocompatibility. The PDA coating on BP materials produced good NIR absorption capacity and efficient photothermal conversion efficiency. NIR irradiation of PDA-coated BP material showed a higher temperature rise of 27.1 °C than that of bare BP (24.1 °C). The thermal images were clear, and the tumor site showed a significant increase in temperature [138].

Similarly, DOX-loaded BP nanosheets coated with PDA and PEOz were developed for the chemo-/photothermal therapy of breast cancer. In severe combined immunodeficient (SCID) mice bearing MCF-7 tumors, IR images were taken when the tumor volume reached about 180 mm^3^. The thermographic map and temperature shift were captured by a thermal imaging camera. After 24 h of injection of formulation by the intravenous route, the tumor tissues were irradiated with 808 nm laser for 5 min. The presence of PDA coating helped to significantly increase the temperature [19].

### 7.3. Photoacoustic (PA) Imaging

PA imaging, also known as optoacoustic imaging, is a novel biomedical imaging technique using laser-generated ultrasound. This technique has been developed by merging high-resolution ultrasound imaging with high-contrast optical imaging. The image contrast relies on the optical characteristics, especially the optical absorption of the tissue, but not on its elastic and mechanical properties. As a result, it is more specific than ultrasound imaging, with the capability of identifying lipids, chromophores, and hemoglobin [139]. Furthermore, with multiple wavelengths of laser, information on molecular characteristics of tissues can be collected and utilized for the determination of in vivo biodistribution of external agents [140].

Sun et al. developed TiL_4_-conjugated BPQDs as a contrast agent for PA imaging of breast cancer. Sulfonic ester of titanium ligand (TiL_4_) was conjugated with BPQDs via surface coordination to enhance the aqueous stability and to use as a PA imaging agent for the in vivo tumor bioimaging. They checked the photothermal efficiency of TiL_4_@BPQD at different wavelengths of NIR and observed reduced PA signal intensity as they increased the wavelength from 680 to 808 nm. Thereafter, they compared the PA performance of formulation with Au nanorods (AuNRs), which have been used as a PA contrast agent. The results indicated that TiL_4_@BPQD is 7.29 times intense than AuNRs at 680 nm at the same concentration. In vitro evaluation in MCF-7 cells showed increased intensity of PA signals (at 680 nm) with the addition of TiL_4_@BPQD. At 12.5 ppm of TiL_4_@BPQD, significant signal was observed, suggesting excellent sensitivity of the formulation for PA imaging. In vivo studies in MCF-7 tumor-bearing Balb/c nude mice showed an increase in PA signals in the tumors (maximum intensity at 4 h after injection). In vivo and in vitro studies thus confirmed the effectiveness of the formulation to be utilized as a PA imaging agent [141]. In another research, PEI- and dextran-modified BPNPs were functionalized with cyanine 7 and folic acid and tested in 4T1 tumor-cell-bearing mice. When PA images were captured at a wavelength of 680 nm, they observed an increase in PA signal with the rise in BPNP concentration from 0 to 200 µg/mL. The formulation showed significant photothermal stability and photothermal conversion efficiency, suggesting that it can be considered an efficient candidate for PA imaging [97]. Similarly, PEGylated BPNPs were formulated to intensify the PA signal with the concentration of the formulation. The tumor-bearing mice were injected with BPNPs, and the PA signal in the tumor region, kidney, and liver were significantly intense compared with preinjection signals. The disappearance of PA signals from the kidney and liver after 24 h of injection indicated effective metabolism of the formulation. The prepared formulation was found to be an excellent PA agent for the clinical diagnosis of breast cancer [48].

### 7.4. Imaging with Radionuclides

**Figure 5:**
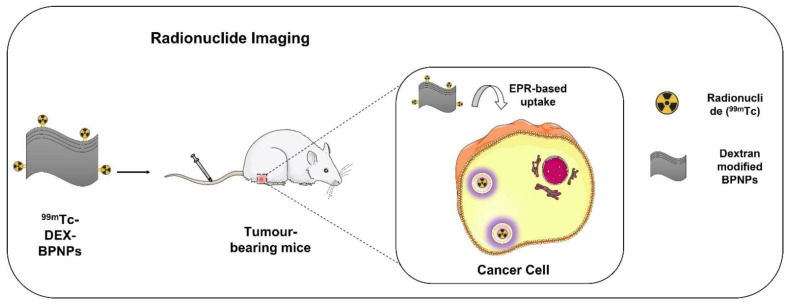
Radionuclide imaging of breast cancer cell using dextran-modified radiolabeled BP nanoparticles.

As phosphorus is present abundantly in the body, it is very challenging to explore the biodistribution, pharmacokinetics (PK), and quantification of BPNPs using methods such as inductively coupled plasma optical emission spectrometry (ICP-OES). Molecular imaging techniques, such as fluorescence imaging, are reported to monitor and quantify the biodistribution of BPNPs by assessing the dynamic change in fluorescence intensity using NIR imaging [62,63]. However, due to the fact that BPNPs can partially quench the fluorescence intensity, alternative approaches are necessary to evaluate the biodistribution of BPNSs. In this context, scintigraphy imaging using radiolabeled probes is a promising option. Dextran-modified BPNPs (DEX-BPNPs) radiolabeled with the radioisotope technetium-99m (^99m^Tc) (Figure 5) were developed, which, due to suitable gamma emission of 140 KeV, helped in quantifying the biodistribution and PK of BPNPs [142]. ^99m^Tc-BP-DEX NPs could be radiolabeled with 83.8% yield and were found to be stable in serum and saline. The nanoconstructs evaluated in 4T1 cells showed cell viability above 80% at a concentration of 100 µg/mL, indicating good biocompatibility. The evaluation of the circulation time of the nanoconstructs in blood showed that they followed a typical two-compartment model, and the DEX-BPNPs have a prolonged blood circulation time owing to their small particle size. The biodistribution and clearance were studied using SPECT/CT imaging for up to 8 h. The maximum accumulation of NPs was found in the liver (35%), retained there for up to 3 h. In other organs, such as spleen and bladder, the accumulation steadily increased from 5% to 20% over a period of 8 h. The formulation was excreted through the hepatobiliary pathway. While these studies are preliminary, further work with preclinical tumor models should indicate the potential for use of these formulations in tumor targeting.

Similarly, Cu^2+^-loaded BPNSs were formulated as efficient photothermal platforms for positron emission tomography (PET)–guided combination treatment of tumor. A strategy to enhance the photothermal stability of BP nanomaterials by conjugating them with Cu^2+^ was proposed as Cu ions can act as nanocaptors of BP, and Cu-based materials show efficient photothermal activity (PTA) and have a synergistic effect on the degradation and photothermal activity of BP. BP@^64^Cu was coated with an RGD-PEG composite for better tissue-specific localization and improved tissue internalization. BPNSs were labeled with ^64^Cu via a chelator-free technique by mixing 100 µL of ^64^CuCl_2_ (370–740 MBq) with 100 µL of BPNS for 1 min. An excellent labeling efficiency of 99% and a molar activity of BP@^64^Cu of ~74 MBq/µg were achieved. The stability of the developed radiolabeled formulation (3.7 MBq) was evaluated using radio-thin-layer chromatography (TLC) in mouse serum and phosphate-buffered saline (PBS). In PBS, the radiolabeled complex showed less degradation (<5%) in 70 h compared with mouse serum, wherein the degradation increased with incubation time with a half-life of 35 h. In vivo PET imaging results demonstrated that the radiolabeled nanoformulation exhibited a long retention time in cancer tissues of about 8% ID/g at 40 h after injection. Ex vivo biodistribution studies showed higher radioactivity in the spleen and liver and, finally, in the lung with the highest tumor uptake of 9.3% ID/g of tissue. A rapid rise in temperature up to 56 °C and improved tumor growth inhibition were noticed in animals injected with radiolabeled BP and irradiated with NIR radiation. The efficient antitumor effect could be attributed due to the better uptake of nanoformulation by the tumor tissues as well as efficient photothermal activity. Further, the therapeutic efficacy of BP@Cu@PEG-RGD was confirmed with PET imaging, where mice treated with the formulation showed reduced tumor size compared with mice treated with saline [114]. The development of radiolabeled BP nanomaterials might be a promising option to achieve multimodal imaging and better therapeutic outcome.

## 8. Strategies for Treating Breast Cancer Using BP Nanomaterials

In comparison with other 2D materials, BP nanomaterials have gained prominence due to their remarkable structural and physicochemical properties. They have been identified as an effective choice for a variety of biomedical applications owing to their unique properties, such as high biocompatibility, superior in vivo biodegradability, and low cytotoxicity. In this section, we concentrated on some of the most relevant therapeutic strategies using BP nanoplatforms for breast cancer management, such as targeted drug delivery, codelivery, gene delivery, PTT, PDT, sonodynamic therapy, reversing the drug resistance, and immunotherapy.

### 8.1. Delivering Chemotherapeutic Agents

Breast cancer therapy includes endocrine therapy, cytotoxic chemotherapy, and cytotoxic chemotherapy + HER2-directed therapy [10]. The most regularly used chemotherapeutic agents for the therapy of breast cancer include doxorubicin (DOX), an anthracycline derivative, and paclitaxel (PTX), which belongs to the taxane family [143]. The major limitations of conventional therapy include nonspecific targeting causing significant side effects, including organ damage. Some chemotherapeutic agents become washed out from the circulation due to engulfment by macrophages. It reduces their circulation time, making them therapeutically ineffective. Multidrug resistance is another drawback of chemotherapeutic drugs. These agents are mostly hydrophobic and hence show poor aqueous solubility and bioavailability [12,13]. Surface modification of BP materials with appropriate polymers and targeting ligands is being explored to overcome problems with conventional chemotherapeutic agents. It is reported that BP nanomaterials can load drugs that weigh more than the carriers; therefore, they are considered good candidates as drug carriers for chemo-photothermal treatment [69].

The presence of phosphoric acid makes the BP surface negatively charged, which can be utilized to load positively charged NPs or chemotherapeutic agents via electrostatic interaction. There are reports on the use of BP nanomaterials for the delivery of chemotherapeutics in tumor therapy due to its large surface area and photothermal efficiency; in bone therapy as phosphorus, which is an important component of the bone; in neurodegenerative diseases; and in implants [76]. Li et al. fabricated a novel approach for the management of triple-negative breast cancer (TNBC) by loading cisplatin into a BP nanosheet, which is then surface-modified with PDA and hyaluronic acid for the controlled release of cisplatin and tumor growth inhibition. Low pH (tumor), hydrogen peroxide, and NIR irradiation, together with the breakdown of BP, were among the external and internal stimuli present in the tumor microenvironment that caused the release of cisplatin. Studies on the prevention of metastasis in 2D monolayers and 3D organoids showed that 4T1 cell migration, invasion, and regrowth were greatly reduced after the administration with nanoformulation combined with NIR light irradiation. Additionally, TNBC-bearing animals administered with BP nanoformulation systemically demonstrated increased tumor cisplatin accumulation, light-triggered suppression of tumor growth, reduced toxicity, and lung metastasis [144]. Generally, the delivery of chemotherapeutic agents with BP nonmaterial is nontoxic to the normal cells and can overcome the limitations of conventional chemotherapy.

### 8.2. Gene-Therapy

Gene therapy is a targeted approach to treat different ailments by using nucleic acid polymers as therapeutic agents. Here, genome is inserted with a functional gene specifically replacing the mutant gene causing abnormalities [145]. However, nucleic acid polymers exhibit poor cellular uptake, possess unstable structure, and are negatively charged. Due to the above properties, they need suitable carriers to avoid degradation [146]. The two approaches available for gene delivery are viral and nonviral systems. Even though the viral vectors are efficient in gene transfer, they exhibit some disadvantages, such as host immune response and high cost [147]. Compared with viral vectors, the synthesis of nonviral vectors is easy and has a negligible risk of adverse immune response in hosts. However, its transfection capability is low due to intracellular and extracellular barriers [148]. Recently, many nanocarriers have been developed, including carbon nanotubes, liposomes, and other 2D materials. Most of them exhibit limitations, such as reduced biodegradation and poor biocompatibility, leading to toxicity. The usage of BP nanomaterials as nanocarriers could overcome these limitations [149,150,151].

Small interfering RNA (siRNA) has received wide recognition due to its applications in gene therapy. siRNA controls the gene expression by limited translation of mRNA by degrading it after the transcription step. Its application in gene therapy is limited due to its poor in vivo cellular uptake and enzymatic degradation in the serum. Effective nanocarriers are needed, which are capable of carrying the genetic material to the target with enhanced cellular uptake. A novel gene delivery system of BPNSs functionalized with PEI loaded with siRNA was developed by Wang et al. The results suggested that the BP-PEI could prevent degradation of siRNA by enzymes, thereby enhancing gene transfection efficiency and bioavailability of siRNA. Stronger fluorescence intensity observed in MCF-7 cells treated with Cy5-siRNA-loaded BP-PEI indicated the enhanced siRNA internalization by the BPNSs. PEI could enhance the escape of siRNA from endosome for successful gene silencing by “proton sponge effect” in vivo. Furthermore, in the in vitro cytotoxicity studies performed on MCF-7 cells, it was observed that the BP-PEI-siRNA could inhibit the growth of cells up to 44% without irradiation and 64% with irradiation of 808 nm NIR. These results indicated that this system can be employed as a synergistic approach, by using both gene therapy and photothermal therapy, in treating the cancer [152].

Mcl-1 is a member of the B-cell CLL/lymphoma 2 (Bcl-2) group, a potential target for cancer treatment, as it shows resistance to chemotherapy. Mcl-1 amplification was also observed in breast cancer cells. BP nanomaterials functionalized with PLL for Cas13a/crRNA delivery were developed to specifically hinder transcription of Mcl-1 for breast cancer treatment. The formulation entered into the cytoplasm by endocytosis and escaped from the endosome via the destruction of BP material. They observed downregulation of Mcl-1 expression of about 58.64% and cell activity inhibition in the in vitro studies performed on AGS cells. The developed BP system has shown its potential application for gene delivery [117].

### 8.3. Codelivery of Drugs

Codelivery of drugs is a widely accepted clinical practice of combining two or more chemotherapeutical agents in a single platform/formulation. This approach shows better clinical potential as the multiple drugs show synergistic effect and target cancer cells via different mechanisms for treatment. Dual drug delivery is reported to show an efficient therapeutic response by inhibiting tumor cell proliferation and reduced toxicity compared with monotherapy [153]. For instance, pH-sensitive dual drug-loaded PEOz functionalized BPNSs were developed for breast cancer chemo-/photothermal therapy. DOX and bortezomib (BTZ) were loaded onto the surface of the BP nanosystem. This codelivery drug platform could deliver two drugs simultaneously to the tumor region. The prepared formulation was capable of releasing BTZ in the acidic microenvironment to produce antitumor activity, and a notable variation in the release of DOX was observed between acidic and basic conditions. At pH 5, about 30% of DOX was released, but at pH 7.4, it was only 11%. An in vitro photoresponsive drug release study revealed the efficient release (95%) of BTZ from the DOX-loaded BP nanoformulation on NIR irradiation for 6 min. There was no drug leakage before reaching the target during the showing complete localization of the drug at the tumor site. Cell viability assay demonstrated that the antitumor activity of the drug was improved with this efficient pH-stimulative NIR-triggered drug delivery system. The study showed the improved activity and specificity of the drugs via a codelivery approach and proved the efficacy of PEOz for precise targeting and prolonged in vivo circulation [19].

### 8.4. Photothermal Therapy (PTT)

Photothermal therapy gained wide attention in the tumor eradication and research field. Usually, the photothermal agents become localized in the tumor site, and light irradiation generates heat and causes cell death by hyperthermia [154,155]. PTT is an efficient strategy to kill cancer cells by generating hyperthermia with minimal side effects. Extinction coefficient (ɛ) and PCE (η) are the two crucial parameters that directly affect the efficiency of PTT. The light absorption capability of a material is denoted by the extinction coefficient, and PCE is the ability to produce heat on exposure with light irradiation. Photothermal materials, such as black phosphorus, graphene oxide, and carbon nanotubes, can potentiate photothermal conversion performance [156].

A low depth of penetration and less photothermal conversion efficiency make PTT less useful in biomedical applications. The use of photosensitizers showing higher absorption in the NIR region and precisely absorbed by the breast tumor cells instead of normal cells leads to selective heating of cancer cells [157,158]. As BP nanomaterials show efficient NIR and ultraviolet absorption and outstanding photothermal conversion property, they can be utilized as photothermal agents for tumor treatment. PEGylated BPQDs were developed, which showed concentration-dependent photothermal activity on irradiation with 808 nm NIR laser and also exhibited outstanding photothermal stability. The in vivo photothermal property of PEGylated nanomaterial was evaluated in 4T1-tumor-bearing mice. Followed by intratumoral injection of formulation, the animals were exposed to NIR radiation using a power density of 2.0 W/cm^2^. Within 5 min of irradiation, the temperature was raised up from 34 to 59 °C. On injection, without NPs, it showed an increase in temperature by only 6 °C. Three days of treatment (PEGylated BPNPs and NIR irradiation) showed notable tumor size reduction, which indicates that BP nanomaterials can be employed as an effective in vivo photothermal agent for the therapy of breast cancer [48]. The photothermal efficiency of FA-conjugated BPQDs was studied by Luo et al. and found that the formulation possesses efficient photothermal stability [79]. Su et al. performed in vivo and in vitro performance of a BP nanosheet–polypyrrole nanocomplex. An in vitro study was performed by incubating 4T1 cells with the formulation, which resulted in 97% killing of tumor cells. In vivo PTT was performed in tumor-bearing mice by using a thermal imaging camera. The temperature of the tumor region rose to 50 °C after 2 min of NIR irradiation. These results demonstrate their potential for treating breast cancer [159].

### 8.5. Photodynamic Therapy (PDT)

PDT differs from PTT in terms of the production of ROS by the photosensitizer platform on irradiation. Recently, PDT drugs, such as porphyrins and their derivatives, have received wide attention due to their good cellular affinity and reduced toxicity [160]. As BP nanomaterials show excellent absorption in the range of visible-light and NIR region, they can produce singlet oxygen (^1^O_2_) on exposure to light of the desired wavelength. It was confirmed by the probe reaction of 1,3-diphenyl-isobenzofuran (DPBF) with ^1^O_2_, resulting in reduced absorption intensity at 410 nm. A cell viability study on human breast cancer cells (MDA-MB-231) exhibited appropriate induction of cell death on irradiation with 660 nm for 10 min. Dichlorofluorescein (DCF), a cell-permeable fluorescent dye, was used to confirm the production of ROS in cells treated with BP nanoformulation and NIR irradiation. The cells produced intense DCF fluorescence and proved ROS production. A cell apoptosis study demonstrated a significant increase in cell apoptosis due to ROS produced during BP-based PDT. Intratumoral injection of BP nanoformulation along with light irradiation resulted in excellent tumor suppression. Terminal deoxynucleotidyl transferase (TdT) dUTP nick-end labeling (TUNEL) assay and immunohistochemical proliferating cell nuclear antigen (PCNA) techniques confirmed the activity of BP nanoformulation as inorganic photosensitizers for breast cancer treatment [161]. Zhang and coworkers also used DPBF as a singlet oxygen probe to detect PDT efficiency. The treatment with BP formulation along with laser irradiation resulted in efficient ^1^O_2_ production. Irradiation with 635 nm laser was found to be better than with 808 nm [162].

In another study, BPNSs were formulated covalently grafted with fullerene C_60_ to potentiate photodynamic therapy. The in vitro photodynamic efficacy of BP-C_60_ was checked on 4T1 tumor cells. The tumor-killing ability of the formulation was increased under NIR light irradiation due to the generation of ROS. A cell viability of about 90% was detected in the absence of irradiation, suggesting that the BP-C_60_ toxicity itself is low. In vivo antitumor activity of BP-C_60_ nanoformulation was determined in a 4T1-tumor-bearing mouse model. BP-C_60_ showed an excellent antitumor effect [163]. Similarly, BPNSs loaded with DOX micelles were developed and incorporated into thermoreversible hydrogel for chemo-photodynamic therapy. The broad absorption spectrum given by the UV–VIS spectroscopy confirmed the potential of a BP nanosheet in PDT as a photosensitizer. The formulation showed less absorbance than that of BPNSs, which confirmed the role of PDT [164].

### 8.6. Sonodynamic Therapy

The therapeutic strategies discussed so far are associated with one or other limitations. For instance, the application of PDT is limited for the superficial sites due to the limited tissue penetration of the light employed. Sonodynamic therapy employs ultrasound, which can penetrate to deep tissue targets and also is considered safe to image even a growing fetus. However, there is a limited availability of the sensitizers used for sonodynamic therapy, and the available sensitizers are unstable when exposed to ultrasound. To address this limitation, Li et al. [165] developed piezoelectric materials as sonodynamic sensitizers and demonstrated the applicability of this concept using BPNSs model. The nanosheets were developed using ultrasonic exfoliation, and the TEM images revealed that the lateral dimension was 183.4 ± 90.2 nm. The noncentrosymmetric structure of BPNSs renders it with the piezoelectric features, and the efficiency of this effect on the developed nanosheets was confirmed using piezoresponse force microscopy. Upon exposure to the ultrasound, BPNSs undergo piezoelectric polarization, inducing a tilt in the energy bands, which after a cascade of reactions leads to the generation of ROS. To test this hypothesis, dichlorofluorescein was used as a fluorescent probe to identify the generation of ROS. It was found that the fluorescence intensity was increased when the BPNS dispersion was exposed to ultrasound excitation, indicating the production of ROS. From further studies, it was also found that the nanosheets could generate ROS even when the dissolved oxygen molecules were not present. This enhances the efficacy and applicability of the nanoconstructs in the hypoxic tumor microenvironment. In vitro cytotoxicity studies performed using CCK-8 assays on 4T1 cells revealed that the ultrasound-exposed nanosheets exhibited significant cytotoxicity by reducing the viability of cells to less than 40% even at a dose of 6.25 µg/mL. Furthermore, the efficacy of the nanosheets to suppress the tumor growth was also confirmed from the 4T1 tumor-bearing mice models.

**Figure 6:**
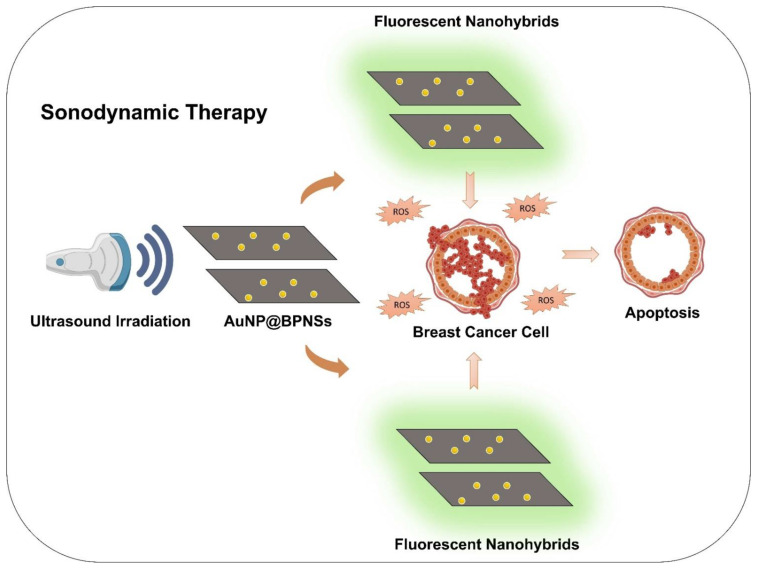
Sonodynamic therapy of breast cancer using AuNP-BPNS nanohybrid.

Similarly, in another study [166], a nanohybrid of gold nanoparticles (AuNPs) and BPNSs were used to develop the sonosensitizers, wherein AuNPs were grown in situ on the surface of BPNSs (Figure 6). The singlet oxygen generation from the nanohybrids when exposed to ultrasound irradiation was estimated using a probe made of singlet oxygen sensor green. It was observed that the increase in fluorescence intensity by the nanohybrids was 313% when compared with the BPNSs, which were near 30%. The study also compared the efficiency of ROS production in the presence of NIR with that of ultrasound irradiation, and the outcome revealed that the ROS generation was 4.7 times higher in the samples exposed to ultrasound irradiation. Further, in vivo studies performed on 4T1 tumor-bearing mice indicated that the nanohybrids could efficiently inhibit the growth of tumor. Hence, sonodynamic therapy has the potential to conquer the limits of the earlier mentioned therapeutic approaches and can find widespread application in the biomedical field.

### 8.7. Combined Phototherapeutic Strategy

Enhanced therapeutic efficiency and reduced side effects are the foremost goals of combining treatment strategies. As BP nanomaterials exhibit excellent surface area, they can carry a good quantity of therapeutic agents within them similar to other 2D nanomaterials and zero dimensional mesoporous materials. Drug delivery along with PTT efficiency and ROS production can be developed for synergistic chemotherapy, PDT, and PTT. Chen and coworkers experimented with the synergistic effect of DOX-loaded BPNSs under 808 and 660 nm of NIR irradiation for combination therapy. The experimental data revealed tumor growth suppression in mice after the combined therapy, exhibiting improved therapeutic efficacy compared with control groups. The effect of combined therapies was further confirmed by collecting tumor tissues of mice for histological examination and confirmed the excellent in vivo antitumor therapeutic effect. These results suggest the therapeutic potential of BPNSs for the therapy of breast cancer [62].

**Figure 7:**
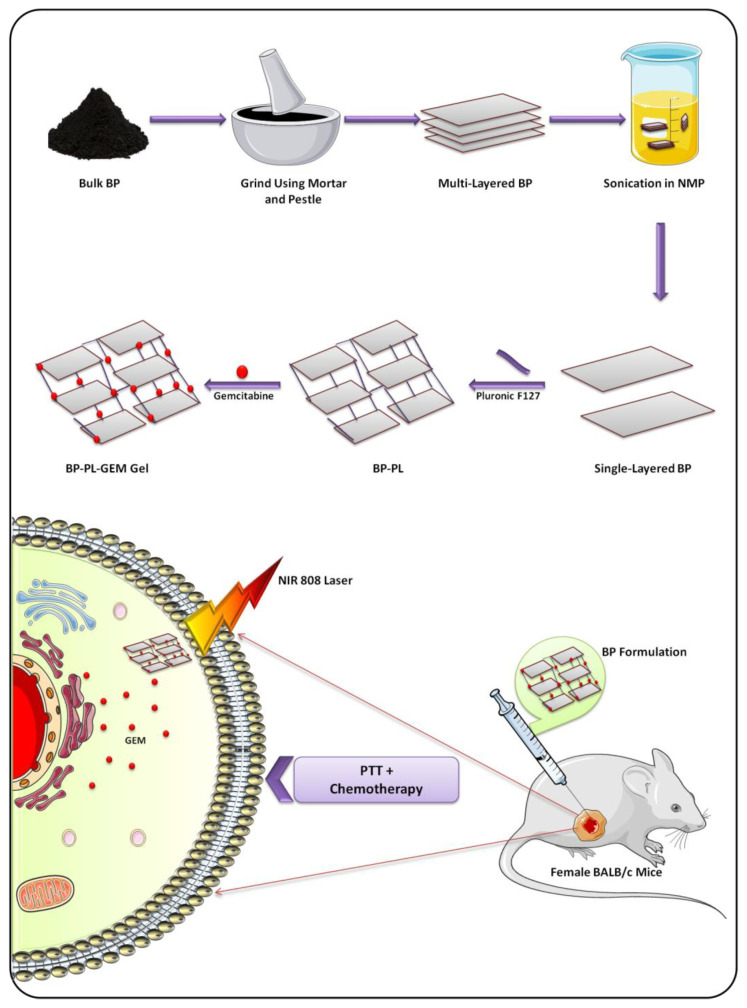
Loading of GEM to the BP nanosheets and combined phototherapeutic activity of the formulation.

Gemcitabine (GEM)-loaded BPNSs conjugated with thermosensitive hydrogel was developed in another research for combinatorial photochemotherapy. The in vivo photothermal activity of the formulation was checked in 4T1 tumor-bearing mice by intratumoral administration. These mice showed a notable rise in temperature of about 70.1 °C after irradiating with 808 nm NIR laser. The chemotherapy with the formulation and with GEM-free drug without NIR irradiation showed partial inhibition of breast tumor growth. PTT/chemotherapy using the formulation and NIR laser irradiation showed potent antitumor activity, as shown in Figure 7. Significant temperature rise and sufficient drug concentration at the tumor site resulted in efficient therapy of cancer [167]. Similarly, BPNSs conjugated on PAMAM-loaded DOX was developed, and a cell viability study on 4T1 cells demonstrated that 20.65% were viable after being treated with a BP nanosheet and NIR irradiation. The study showed outstanding photothermal efficiency by potentiating cancer cell apoptosis as a photothermal agent. The formulation without NIR irradiation showed reduced cytotoxicity. In vivo antitumor activity was ascertained in 4T1-tumor-bearing mice. The BP nanoformulation with NIR irradiation potentiated the drug release and photothermal conversion on 808 nm NIR irradiation. There was no remarkable difference in body weight after various treatments [100]. Table 2 describes the different applications of BP nanoplatforms for phototherapy.

### 8.8. Reversing Drug Resistance

Even though chemotherapy is the extensively used and effective modality for cancer treatment, drug resistance can cause treatment failure. Multidrug resistance (MDR) is observed in tumor cells against many drugs. Some of the molecular mechanisms behind this process include over expression of P-gp transporter, increased detoxification activities, poor drug uptake, and DNA repair activation. These escape characteristics gained by the drug-resistant cells result in converting chemotherapeutic agents into inactive derivatives with diminished anticancer activity [170]. A study conducted by Wu and coworkers focused on developing BP nanoformulation loaded with phenyl isothiocyanate (PEITC) for reversing resistance breast cancer cells. The mutant gene p53 present in the breast cancer cells suppresses the specific target genes and potentiates antiapoptosis properties to withstand chemotherapeutic agents such as DOX. The higher dose of DOX is also not effective to inhibit tumor growth and can cause serious side effects on normal cells. In such cases, a treatment strategy should be to target the mutant p53 gene to reduce MDR. PEITC can selectively deplete the mutant p53, and the nanodrug delivery system incorporated with PEITC can be used as an effective strategy to reverse MDR. An in vivo antitumor efficacy study in tumor mice models showed that drug-loaded BP nanoformulation without PEITC showed a slight reduction in the tumor due to drug resistance in tumors. The PEITC-loaded BP formulation exhibited better antitumor activity due to the reversal of MDR. Tumor growth inhibition was potentiated by treating tumor-bearing mice with the formulation along with NIR laser irradiation [82].

Similarly, another research work focused on developing a novel multifunctional BP nanosheet-based codelivery system for chemo-/photo-/gene therapy to reverse MDR. DOX and P-gp siRNA were loaded in the BPNSs, wherein P-gp helps in downregulating P-gp expression on the tumor cell membrane, which is responsible for MDR. An in vitro cytotoxicity study showed excellent cytotoxicity due to the abolition of P-gp-mediated drug resistance by the P-gp siRNA-loaded formulation. The in vivo antitumor efficiency of the prepared formulation was studied in a subcutaneous xenograft tumor model. Gene–chemo combined therapy groups showed better inhibition activity than the chemotherapy group, this could be due to the inhibition of drug resistance by P-gp siRNA. The prepared formulation could transport P-gp siRNA and DOX to the tumor site and obstruct MDR [138].

### 8.9. Immunotherapy

Cancer immunotherapy, which may either initiate or augment the host immune response in order to identify and target tumor cells, has received a lot of interest. Many immunotherapy techniques have recently been employed in clinical research to manage cancer metastasis, which includes monoclonal antibody (mAb) treatment, tumor vaccination, cytokine therapy, checkpoint blocking, and chimeric antigen receptor (CAR) T-cell therapy [171]. Many tumors, however, are immunosuppressive by nature and are difficult to treat with existing immunotherapy strategy. Combining phototherapy with immunotherapy can effectively circumvent these limits while also enhancing the therapeutic efficacy, which might not only preserve the benefits of immunological techniques, such as long-term anticancer immunity and systemic immune activation, but also enhance the selectivity of the entire therapy [172]. As a result, an approach known as photoimmunotherapy (PIT), which combines phototherapy with immunotherapy, has evolved to meet the objective of eliminating primary tumors and reducing the metastasis. PIT is a synergistic therapeutic method that enhances the benefits of both phototherapy and immunotherapy while minimizing their inherent drawbacks. This combination therapy approach can not only remove the original tumor and clear any remaining tumor cells, but also track metastatic areas, giving additional options to patients with advanced cancers [173].

In a recent study by Li and his colleagues, they formulated a novel kind of NIR and ROS-sensitive BP nanovesicles (BPNVs) loaded with CpG oligodeoxynucleotide, an immunoadjuvant for photodynamic immunotherapeutic treatment against cancer development. CpG is a strong immunoadjuvant that boosts cytokine release by antigen-presenting cells (APCs) and have shown promising activity in clinical trials. BPNVs were formulated by grafting BPQDs with PEG and poly (propylene sulfide) (PPS) that act as an ROS-sensitive part. When it reaches the tumor site, high quantities of ROS are produced by BPNVs in response to NIR laser irradiation, causing the hydrophobic polymer PPS to shift to hydrophilic in nature, resulting in vesicle breakdown and formation of BPQD. The encapsulated CpG was then released into the tumor site, where it was collected together with the tumor antigens by the APCs, which were then activated to secrete numerous cytokines that boosted the antitumor immune response. In vivo testing on 4T1 tumor-bearing BALB/c mice revealed that BPNV-CpG-mediated photoimmunotherapy not only suppressed tumor cell proliferation, but also prevented tumor development and metastasis to distant sites. The immune response of the nanoformulation was evaluated with enzyme-linked immunosorbent assay (ELISA), where the results indicated that the serum levels of IL-6, TNF-α, and IL-12 were notably higher in BPNV-CpG treated animals compared with other groups. This result indicated that administering BPNVs-CpG with NIR light irradiation greatly increased immune response, which could be due to the combined action of photodynamic-induced immunogenic cell death (ICD) and CpG stimulation [174]. Yue et al. reported a BP-based nanoplatform for the delivery of Cas13a/crRNA for the therapy of breast cancer. The developed nanosystem could inhibit Mcl-1 during transcriptional stage, thereby downregulating Mcl-1 expression and enhancing breast tumor cell apoptosis [117].

PTT is an efficient approach for producing an in situ tumor neoantigen that has a lot of potential in tumor immunotherapy. Liang and colleagues reported a biomimetic BPQD formulation to use NIR laser irradiation to trigger breast cancer cell death in situ and activate the immune system to eradicate metastatic and residual tumor cells via a combination with the PD-1 antibody (aPD-1) administration. BPQDs were coated with the erythrocyte membrane (RM) to form biomimetic nanovesicle of BPQD (BPQD-RMNV), which showed efficient in vivo tumor accumulation and prolonged circulation. In vivo results suggested that the cancer cells exhibited apoptosis after being exposed to NIR laser light, resulting in the recruitment of dendritic cells to collect the antigens released. Following that, a strong tumor-specific CD8^+^ T cell response was elicited both at the primary tumor site and at the metastatic tumor location. PTT induced by BPQD-RMNV in combination with aPD-1 treatment dramatically reduces primary and secondary tumor development [83]. Zhao and team developed a novel approach by coating BPQDs with a cell membrane for tumor-specific PTT. They found that the developed system can kill cancer cells via photothermal effect and also induces the dendritic cell maturation. They combined BPQDs with αPD-L1, which can improve T-cell immune response and thus increases recognition and tumor cell killing efficiency by the T cells. In addition to effectively treating distant cancers by reprogramming the immunosuppressive TME and enhancing local and systemic anticancer immune responses, the PTT and PD-L1 immunotherapy combo also prevents tumor spread via the immunological memory effect. This combined therapy resulted in an effective inhibition of recurrence in triple negative breast cancer [111]. In a similar study by Zhang and colleagues, fabricated PEGylated hyaluronic acid modified BP NPs for combinational PTT, PDT, and PIT. In vitro results indicated that the developed formulation caused the downregulation of CD206 expression by 42.3% and the upregulation of CD86 by 59.6%, which suggested the effect of BP nanoformulation in the remodeling of tumor-associated macrophages. In vitro and in vivo studies demonstrated that combining PDT, PTT, and HA-BP immunotherapy could not only efficiently restrain original tumor but also induce ICD and release damage-associated molecular patterns (DAMPs), which could enhance dendritic cell maturation and effector cell activation, which could robustly elicit antitumor immune responses for tumor therapy [162].

## 9. Degradability and Toxicity of BP Nanomaterials

Currently, the toxicity of BP nanomaterials in the biological system is a big concern. BPNSs with immense lateral size and thickness show highest toxicity, while the smallest BPNSs exhibit only modest toxicity. Chemical modification or functionalization of BP can result in a significant reduction of toxicity [175]. Intravenous injection of pristine BP into mice generated an inflammatory response. The toxicity of BPNSs has some advantages, as it kills bacteria by oxidative damage and by destroying the bacterial membrane. Even though a report on the biological effects of BPNSs is available, their cellular-level toxicity mechanism is not clear, and in vivo biosafety has scarcely been reported [176].

It is essential to evaluate the biosafety and biodegradability of nanomaterials to confirm the safety of biological tissues. BP nanomaterials exhibit excellent biocompatibility and are suitable for biomedical applications. Phosphorus is a bone constituent and makes ~1% of the total body weight. In BPNSs, the sheets are not flat, and elemental atoms are connected by weak van der Waals force. It makes them able to react with water and oxygen and terminally degrade to nontoxic phosphate and phosphonate in the biological fluid, which makes them preferable when compared with other two-dimensional materials, which accumulate in the organs [177]. A degradability study of BP nanomaterials (QDs) was conducted by Shao and team using hydrophobic PLGA. In vitro cytotoxicity of the BPQDs/PLGA was examined on MCF7 and B16 (melanoma cells) using CCK-8 assay. Even at a higher concentration of BPQDs (100 p.p.m.), no significant cytotoxicity was observed in all types of cells. MCF7 cells bearing BALB/c nude mice were employed for an in vivo study. The injection dose of the prepared BP nanoformulation was 10 mg BP kg^−1^, and histological, biochemical, and hematological analyses were performed at 1, 7, and 28 days after injection. The major hematological markers, such as red blood cells, mean corpuscular volume, corpuscular hemoglobin concentration, and hematocrit, were calculated. Compared with control groups, the treated group showed all parameters in the normal range, and the difference was not statistically significant (*p* > 0.05). These results demonstrated that the prepared BP nanoformulation has no considerable inflammation and infection in the treated mice. The BP-treated mice were exposed to 24 h of artificial daylight illumination, and no significant phototoxicity was observed, which is generally observed with many photosensitizer molecules. The system had a better clinical potential with promising PTT efficiency along with combined biodegradability and biocompatibility [63].

A research team performed in vivo and in vitro toxicity studies of BPNSs and found that reduction in the viability of cells was dependent on time and dose in the in vitro toxicity studies. An increase in intracellular ROS was observed due to the interference of BPNSs with mitochondrial membrane potential. They performed a toxicity study of BP nanomaterials on L929, 4T1, and A549 cell lines by 3-[4,5-dimethylthiazol-2-yl]-2,5-diphenyltetrazolium bromide (MTT) assay. Even at a high concentration of BP (200 µg/mL^−1^), no toxicity was observed in four cell types. The in vivo studies were carried out on mice bearing 4T1 tumor. No toxicity was observed in mice by a single injection of BPNSs, while a toxic effect was observed on multiple doses of BPNSs inducing renal and hepatic toxicity. The renal and hepatic functions reverted to normal after the recovery period, indicating that the BPNSs displayed excellent biosafety [62]. A cytotoxicity study was carried out in MCF7 cells and normal LO2 cells in another study using the standard Cell counting Kit-8 (CCK-8). No cytotoxicity was noticed even at a large concentration of 75 ppm in both cells, which confirmed that they possess excellent biocompatibility and are appropriate for biomedical application [134].

## 10. Future Perspective

BP has good reactivity with oxygen even in the absence of light illumination and was found to follow the pseudo-first-order reaction kinetics. The reaction preferentially occurs from the P atoms at the BP edges, forming the phosphate ions, leading to the structural decay in water. Under this mechanism, the BP aqueous suspension can be stored in a N_2_ bubbled sealed container under ambient light for months or can sustain in oxygenated water for a number of days for applications. It was also supported by the Raman spectra, which confirmed the structural integrity of the BP sample stored under ambient light in deoxygenated water for 15 days [178]. Similar studies were conducted in recent years to know the degradation mechanism of BPNSs in protic solvents and when exposed to atmospheric oxygen [179]. It was found that covering the BP nanoflakes with ionic liquids, such as 1-butyl-3-methylimidazolium tetrafluoroborate (BMIM-BF_4_) [180], or with biocompatible polymers, such as PLGA [63], would enhance the stability of BPNS. Besides, few studies have shown that the drug itself can be used to develop stable BPNSs, foreshadowing the path for its clinical application [69].

Postoperative breast abnormalities are common in patients who have undergone surgical resection, and thus, breast tissue repair is critical for enhancing patients’ quality of life. Therefore, in addition to the complete ablation of tumor cells, therapeutic strategies should also focus on the potential of the nanoconstructs for reconstructing breast tissues. Recently, BP has also found application in the area of regenerative medicine. It was reported in a study that BPNSs in combination with gelatin could not only destroy breast cancer cells but also induce adipose tissue formation [181]. The photothermal characteristics of BP led to the destruction of cancer cells, while the gelatin present in the composite helped in the adhesion and proliferation of the mesenchymal stem cells (MSCs). The in vitro and in vivo studies demonstrated that the composite scaffold could improve lipid oil droplet formation, upregulating the expression of genes responsible for adipogenesis in the MSCs. Such results encourage the researchers to work more in developing multimodal nanoplatforms, which ultimately paves the way for the clinical translation of BP nanoconstructs in different fields of biomedicine. In conditions such as neurodegenerative disorder (ND), dyshomeostasis of transition metals acts as a crucial factor. Excess of such ions can catalyze ROS formation and thereby induce apoptosis of neuronal cells. Novel chelating compounds that can capture excess redox-active metal and traverse the blood–brain barrier are desperately needed for ND treatment. In this context, BPNSs have found their extended application in capturing the common transition metal ions (such as Cu^2+^ and Zn^2+^) in the human body. By functioning as an antioxidant, they can also reduce the cytotoxic ROS formation caused by Cu^2+^ dyshomeostasis [182], resulting in therapeutic efficacy against ND. Similar research studies have to be performed to explore the potential of BP nanoplatforms in various biomedical fields.

## 11. Conclusions

Breast cancer is the prevailing malignancy in women worldwide. Conventional cancer treatment approaches have limitations, making personalized medicine an essential strategy for managing breast cancer. With the quick expansion of multidisciplinary nanomedicine, nanosystems with adaptable compositions, structures, and morphologies have been made for diagnosis and therapy, primarily in the fight against cancer. Currently, two-dimensional BP nanosheets have attracted considerable attention and are best suited for theranostic nanomedicine because of their excellent biocompatibility with negligible toxicity, efficient drug loading due to a large surface area, better photothermal conversion efficiency, and high performance in photodynamic cancer therapy. Furthermore, as BP materials possess excellent electrochemical catalytic activity, they can be used as electrochemical biosensors. The major challenge for the biomedical application of BP nanomaterial is to prevent its degradability in water. The unstable structure of BP material will inversely affect the photothermal efficiency and its storage. Various functionalization techniques have been developed to address this and make the formulation stable and suitable for biomedical applications. Biomolecules, such as DNA and siRNA, can also be conjugated to the surface of functionalized BP-based systems for gene transfection. These characteristics make BP-nanomaterial-based drug delivery systems a promising candidate for theranostic applications. With recent breakthroughs and novelties in the realm of 2D nanomaterials, there remains a vision to address challenges and proactively capitalize on upcoming opportunities to broaden the focus on the application of BP in the biomedical field and accomplish clinical translation.

## Figures and Tables

**Table 1:** Important characteristics of BP nanomaterials for biomedical applications.

Properties	Exhibited by BP	Related Biomedical Applications
Bandgap (eV)	0.3–2.0	Broad range of bandgap results in excellent optical absorption along UV, visible, and IR spectrum for the detection of biomolecules, such as proteins and various inorganic ions, which makes BP a right choice for biosensing, phototherapy, and photoacoustic imaging [65].
Electrical conductivity	Ambipolar	As BP is ambipolar, it can detect both positively and negatively charged bioanalytes for efficient biosensing [66].
Carrier mobility (cm^2^·V^−1^·s^−1^)	1000	Excellent carrier mobility exhibited by BP nanomaterials makes them suitable for gas sensing based on electrical conductivity measurement [67].
Biocompatibility	Excellent	BP shows better biocompatibility and comparatively less cytotoxicity [68].
In vivo biodegradability	Excellent	BPNSs can degrade to nontoxic phosphate and phosphonate in vivo, and hence, they do not produce any immune response or have toxic potential [69].
Surface area (m^2^·g^−1^)	~2630	BPNSs exhibit a large surface area with single-atomic thickness and contain a large number of spots for anchoring the chemotherapeutic agents [70]. They can load drugs that weigh more than the carriers; hence, BPNSs are considered good candidates to be used as drug carriers for chemo-photothermal therapy [24,71].

**Table 2:** Applications of BP nanomaterials in phototherapy.

Type of BP Nanosystem	Targeting Ligand	Modification	Therapy	Cell Line	Animal Model	Results	Reference
BPNS	-	PEITC	PTT/PDT/gene therapy	MCF-7/ADR	BALB/c nude mice	Inhibition of drug-resistant cancer	[82]
BPQDs	-	PNIPAM	PTT/immunotherapy	MDA-MB231	BALB/c nude mice	Stimulation of γδ T-cell for cancer immunotherapy	[168]
BPQDs	-	PLGA	PTT	4T1-LG12	BALB/c mice	Apoptosis-dependent tumor cell death	[121]
BPNS	-	Nile blue	PTT	MCF-7	BALB/c nude mice	Tumor ablation under NIR	[134]
BPNS	-	-	PTT/PDT	4T1	BALB/c mice	Enhanced drug release on NIR irradiation	[62]
BPQDs	-	PLGA	PTT	MCF-7	BALB/c nude mice	Better PTT and tumor-targeting efficiency	[63]
BPNS	-	Au	PTT	4T1	BALB/c mice	Enhanced photothermal conversion efficiency	[169]
BPNS	-	PEI	PTT/gene therapy	MCF-7	BALB/c nude mice	Better PTT and tumor-targeting efficiency	[152]
BPNS	-	Polypyrrole	PTT	4T1	BALB/c mice	Efficient performance of NIR PTT	[159]
BPNS	-	PEOz	PTT	MCF-7	Female severe combined immunodeficient (SCID) mice	Targeted long circulation and better cellular uptake	[19]
BPNS	HA	PAMAM	PTT	4T1	BALB/c mice	Better therapeutic effect	[100]
BPNS	HA	-	PTT	4T1	Balb/c mice	pH/NIR-triggered drug release	[80]
BPNPs	FA	DEX	PTT	4T1	BALB/c mice	Excellent photothermal conversion efficiency	[97]

BPNS, black phosphorus nanosheets; BPQDs, black phosphorus quantum dots; BPNPs, black phosphorus nanoparticles; PEITC, phenethyl isothiocyanate; NIPAM, N-isopropylacrylamide; PLGA, poly (lactic-co-glycolic acid); PEI, polyethylenimine; PEOz, poly(2-ethyl-2-oxazoline); PAMAM, poly (amidoamine); DEX, dextran.

## Data Availability

Not applicable.
